# Endothelial ferroptosis in blood–brain barrier dysfunction and neuroinflammation: mechanisms and immune–vascular crosstalk

**DOI:** 10.3389/fimmu.2026.1825000

**Published:** 2026-06-04

**Authors:** Yue Liu, Lei Yin, Peng Zhang, Wangwen Li

**Affiliations:** 1 Department of Encephalopathy, Yunyang Hospital, Chongqing University of Chinese Medicine, Chongqing, China; 2 Department of Neurology, The Southern Yunnan Central Hospital (The First People’s Hospital of Honghe Hani and Yi Autonomous Prefecture), Mengzi, Yunnan, China; 3 Department of Geriatrics, Chongqing University Three Gorges Hospital, Chongqing University, Chongqing, China; 4 Department of Neurology, Chongqing University Three Gorges Hospital, Chongqing University, Chongqing, China

**Keywords:** blood–brain barrier, brain microvascular endothelial cells, endothelial ferroptosis, immune–vascular crosstalk, innate immune sensing, lipid peroxidation, neuroinflammation, tight junctions

## Abstract

Ferroptosis is an iron-dependent form of regulated cell death driven by phospholipid peroxidation. In the central nervous system (CNS), most ferroptosis research has focused on neurons and glial cells, whereas the vulnerability of brain microvascular endothelial cells (BMECs) and its consequences for blood–brain barrier (BBB) integrity remain less clearly defined. Because BMECs form the vascular interface between the circulation and the brain parenchyma, ferroptotic injury in this cell population may represent an immunovascular mechanism through which endothelial redox stress is translated into barrier dysfunction and neuroinflammatory amplification. In this review, we summarize molecular pathways that may promote or restrain BMEC ferroptosis, including iron handling, antioxidant defense mediated by the solute carrier family 7 member 11 (SLC7A11)–glutathione peroxidase 4 (GPX4) axis and nuclear factor erythroid 2-related factor 2 (Nrf2) signaling, lipid peroxidation, and junctional remodeling. We then discuss how ferroptosis-associated endothelial injury may contribute to BBB leakage, damage-associated molecular pattern release, innate immune sensing, leukocyte recruitment, glial activation, and self-amplifying inflammatory feedback at the neurovascular interface. We organize the available literature according to the strength and cellular specificity of evidence, separating BMEC-specific findings, BBB-focused *in vivo* studies, indirect CNS evidence, and mechanistic analogies from non-CNS endothelial systems. Finally, we evaluate disease-specific evidence in ischemic stroke and selected neurodegenerative or inflammatory conditions, together with therapeutic strategies, BMEC-targeting considerations, candidate clinical biomarkers, and translational barriers for modulating endothelial ferroptosis. This review frames endothelial ferroptosis as a promising but incompletely established immunovascular link between BBB dysfunction and neuroinflammation, and highlights the need for BMEC-specific models, human BBB systems, endothelial ferroptosis biomarkers, biomarker-guided monitoring, BMEC-targeted delivery approaches, and careful evaluation of the physiological risks of systemic or prolonged ferroptosis blockade.

## 1 Introduction

Neuroinflammation is a common pathological feature of many central nervous system (CNS) disorders, including acute cerebrovascular injury and chronic neurodegenerative diseases (1, 2). Under physiological conditions, inflammatory signaling contributes to host defense, tissue repair, and restoration of homeostasis; however, persistent or dysregulated neuroimmune activation can disturb neuronal, glial, and vascular function and thereby amplify disease progression (1). The blood–brain barrier (BBB) is a specialized neurovascular interface that maintains CNS homeostasis by regulating molecular exchange and limiting uncontrolled entry of circulating immune cells and inflammatory mediators into the brain parenchyma (3, 4). Brain microvascular endothelial cells (BMECs), together with tight junction complexes and other components of the neurovascular unit, form the structural and signaling backbone of the BBB. Increasing evidence suggests that BMEC dysfunction is not merely a late consequence of CNS pathology, but may occur early and shape subsequent barrier leakage, immune-cell trafficking, and parenchymal inflammation (4, 5).

Ferroptosis is an iron-dependent form of regulated cell death driven by excessive phospholipid peroxidation and failure of lipid peroxide detoxification (6, 7). In the CNS, ferroptosis has been widely discussed in relation to neuronal and glial injury in stroke, neurodegeneration, and other brain disorders (8). By contrast, the role of ferroptosis in BMECs remains less clearly integrated into current models of BBB dysfunction and neuroinflammation, although direct experimental evidence indicates that BMEC ferroptosis can contribute to hypoxia-induced BBB injury (9). This distinction is important because BMECs occupy a unique position between the systemic circulation and the brain parenchyma. Their barrier-forming phenotype, polarized transport systems, exposure to circulating inflammatory cues, and high demand for redox homeostasis may create a cellular context in which ferroptotic stress has consequences beyond endothelial cell loss alone (3, 9).

From an immunological perspective, BMEC ferroptosis is relevant not only because it may weaken the physical barrier, but also because ferroptosis-associated endothelial injury may convert vascular redox stress into immune signaling at the neurovascular interface (10–12). Lipid peroxidation, junctional remodeling, endothelial activation, and damage-associated molecular pattern release may contribute to innate immune sensing, leukocyte adhesion and transmigration, glial activation, and inflammatory feedback (4, 13). Once vascular-derived danger signals or plasma components reach the perivascular space and brain parenchyma, microglia and astrocytes may participate in cytokine production, inflammasome activation, and secondary inflammatory amplification (4, 13). Nevertheless, the extent to which each step is directly caused by BMEC ferroptosis differs across experimental models, and several links remain inferential rather than fully established.

Several previous reviews have summarized ferroptosis in BBB injury, cerebrovascular pathology, or CNS disease more broadly (14, 15). The distinctive aim of this review is therefore not to repeat general ferroptosis mechanisms, but to evaluate BMEC ferroptosis as a potential immune–vascular interface linking BBB dysfunction and neuroinflammation. To improve evidentiary clarity, we distinguish four levels of support throughout the review: BMEC-specific direct evidence, BBB-focused *in vivo* evidence, CNS indirect evidence, and mechanistic analogies from non-CNS endothelial systems. This evidence framework allows established observations to be separated from emerging mechanisms and hypothesis-driven extrapolations.

In the following sections, we first discuss why BMECs may be uniquely vulnerable to ferroptotic stress compared with neurons, astrocytes, and pericytes. We then summarize molecular mechanisms by which BMEC ferroptosis may affect BBB integrity, with particular attention to iron handling, antioxidant defenses, lipid peroxidation, and junctional remodeling. Next, we examine the stepwise immune–vascular cascade through which endothelial ferroptosis-associated injury may promote innate immune activation, leukocyte recruitment, glial responses, and inflammatory feedback. Finally, we evaluate disease-specific evidence, therapeutic strategies, BMEC-targeting considerations, candidate clinical biomarkers, and current translational barriers for modulating endothelial ferroptosis to preserve or restore BBB function.

We conducted a structured narrative literature search in PubMed, Web of Science, and Google Scholar using combinations of the terms “ferroptosis,” “brain microvascular endothelial cells,” “blood–brain barrier,” “neuroinflammation,” “tight junction,” “immune cell trafficking,” “damage-associated molecular patterns (DAMPs),” “stroke,” “Alzheimer’s disease,” and “Parkinson’s disease.” We prioritized studies with BMEC-specific ferroptosis readouts, BBB functional measurements, and immune or inflammatory endpoints. Evidence from non-CNS endothelial systems or broader ferroptosis literature was included only when directly relevant to mechanistic interpretation and was distinguished from BMEC- or BBB-focused evidence.

Taken together, this review treats BMEC ferroptosis not simply as an endothelial cell-death event, but as a potential vascular-to-immune signaling process that may connect BBB structural dysfunction with neuroinflammatory amplification. By applying an evidence-stratified framework, we aim to clarify where BMEC ferroptosis is directly supported, where BBB-level observations remain indirect, and which translational questions must be addressed before endothelial ferroptosis can be considered a therapeutically actionable mechanism.

## 2 Cellular and microenvironmental determinants of BMEC ferroptosis susceptibility

BMECs occupy a specialized position within the neurovascular unit. They are not merely passive structural components of the BBB, but metabolically active, polarized endothelial cells that directly interface with circulating iron, oxygen fluctuations, inflammatory mediators, and hemodynamic forces (9, 16). Compared with neurons, astrocytes, or pericytes, BMECs must simultaneously maintain tight junction integrity, regulate transendothelial transport, and respond to systemic vascular cues. These features may shape a cell-specific threshold at which redox imbalance is converted into lipid peroxidation and ferroptotic injury. The available evidence, however, is not uniform across all proposed mechanisms. Some findings come from BMEC-specific or BBB-focused models, whereas others derive from broader ferroptosis biology, non-CNS endothelial systems, or disease-context observations. Against this background, BMEC ferroptosis susceptibility can be considered across three interrelated dimensions: endothelial iron handling, anti-ferroptotic defense capacity, and the cerebrovascular microenvironment.

### 2.1 Iron handling at the BBB and endothelial iron loading

Iron transport across the BBB is tightly regulated, and BMECs participate in brain iron acquisition through transferrin receptor 1 (TfR1)-dependent and related transport mechanisms (16). This physiological role places BMECs at a critical interface between systemic iron availability and CNS iron homeostasis. Under pathological conditions, increased iron uptake, impaired iron handling, or noncanonical iron loading may expand the endothelial labile iron pool, thereby favoring reactive oxygen species generation and phospholipid peroxidation (17–19). Direct BMEC-focused evidence supports this possibility: hypoxic stress has been shown to induce ferroptosis-associated changes in bEnd.3 cells and zebrafish cerebrovascular endothelial cells, accompanied by reduced claudin-5 (CLDN5) expression and BBB disruption (9). In ischemic brain injury models, rosmarinic acid-loaded liposomes suppressed ferroptosis by inhibiting TfR1 in BMECs, further supporting TfR1-associated iron handling as a relevant endothelial mechanism (12).

Additional studies support the broader concept that BBB iron dyshomeostasis can contribute to vascular dysfunction, although these findings should not be treated as equivalent to direct BMEC ferroptosis evidence. In β-thalassaemia mice, systemic iron overload was associated with cognitive impairment, vascular iron accumulation, reduced zonula occludens-1 (ZO-1) expression, and altered BBB integrity (20). Toxicological evidence also indicates that cadmium exposure can impair brain endothelial barrier function by disrupting iron homeostasis and activating ferroptosis-related pathways (21). In chronic cerebral hypoperfusion-related white matter injury, myelin debris endocytosis by brain endothelial cells was linked to endothelial iron overload and impaired iron availability for oligodendroglial lineage cells (22). Together, these observations indicate that BMECs can participate in disease-related iron redistribution, but further work is required to determine when endothelial iron loading directly triggers ferroptosis rather than other forms of endothelial dysfunction.

### 2.2 Anti-ferroptotic defense systems and the endothelial redox threshold

BMEC vulnerability to ferroptosis is also shaped by endogenous antioxidant systems that restrain lipid peroxide accumulation (14, 23, 24). The SLC7A11–glutathione–GPX4 axis is a central ferroptosis-defense pathway because GPX4 activity limits the accumulation of toxic lipid hydroperoxides (7). In BMEC-focused hypoxia models, ferroptosis-associated BBB injury has been accompanied by lipid peroxidation and reduced anti-ferroptotic defenses, supporting the relevance of this pathway in brain endothelial injury (9). Nrf2-regulated antioxidant signaling may further support endothelial resistance to ferroptotic stress by promoting downstream cytoprotective responses, including heme oxygenase-1 (HO-1) and GPX4-related programs (25–27). In cerebral ischemia/reperfusion models, edaravone dexborneol protected the BBB by inhibiting ferroptosis through activation of the Nrf2/HO-1/GPX4 pathway (26). Conversely, lipocalin-2 aggravated BBB dysfunction after intravenous thrombolysis by promoting endothelial cell ferroptosis through regulation of the high-mobility group box 1 (HMGB1)/Nrf2/HO-1 pathway (28). These BBB-focused studies support the relevance of Nrf2-linked antioxidant defenses, although the precise contribution of BMEC-autonomous Nrf2 signaling may vary by disease model.

Beyond the canonical GPX4-dependent pathway, the ferroptosis suppressor protein 1 (FSP1)–coenzyme Q10 (CoQ10) system has emerged as a GPX4-independent mechanism that suppresses lipid peroxidation by maintaining reduced CoQ10 at cellular membranes (29, 30). This pathway broadens the anti-ferroptotic defense network beyond SLC7A11 and GPX4. However, current evidence for FSP1–CoQ10 in BMEC ferroptosis remains less direct than evidence for GPX4- and Nrf2-associated pathways. FSP1–CoQ10 should therefore be framed as a plausible auxiliary defense mechanism that requires validation in BMEC-specific and BBB-focused systems, rather than as an established BMEC ferroptosis pathway.

### 2.3 Cerebrovascular microenvironment and mechanical regulation of ferroptosis susceptibility

The ferroptotic threshold of BMECs is likely influenced by the local cerebrovascular microenvironment. Oxygen tension, inflammatory mediators, pericyte-derived signals, aging-related endothelial stress, and hemodynamic forces can alter endothelial metabolism, junctional stability, and redox balance. Proteomic analysis of cerebral microvascular endothelial cells under different oxygen conditions suggests that oxygen availability can reshape brain endothelial cellular programs (31). In aging models, hypoxia-induced BBB dysfunction has been associated with increased endothelial permeability and mitochondrial reactive oxygen species generation through arginase-II-dependent mechanisms (32). These findings are relevant to ferroptosis susceptibility because hypoxia and mitochondrial oxidative stress can intensify endothelial redox imbalance, but they should be considered context-setting evidence rather than direct proof of BMEC ferroptosis.

Signals from neighboring neurovascular-unit cells may also modify BMEC injury responses. Pericytes can regulate gene and protein expression in BBB endothelial cells, suggesting that endothelial–pericyte crosstalk may reshape BMEC responses to metabolic and oxidative stress (33). In addition, shear stress can influence transcriptional programs in *in vitro* BBB models, indicating that mechanical cues are relevant to BMEC phenotype and barrier function (34). A recent BBB mechanobiology review further highlights that shear stress, cyclic strain, tissue stiffness, and extracellular matrix composition can influence BBB integrity during development, disease, and aging (35). These microenvironmental and mechanical factors should not be described as direct ferroptosis mechanisms unless ferroptosis-specific readouts are available. Instead, they are best presented as determinants that may modulate BMEC vulnerability to ferroptotic injury.

**Figure 1:**
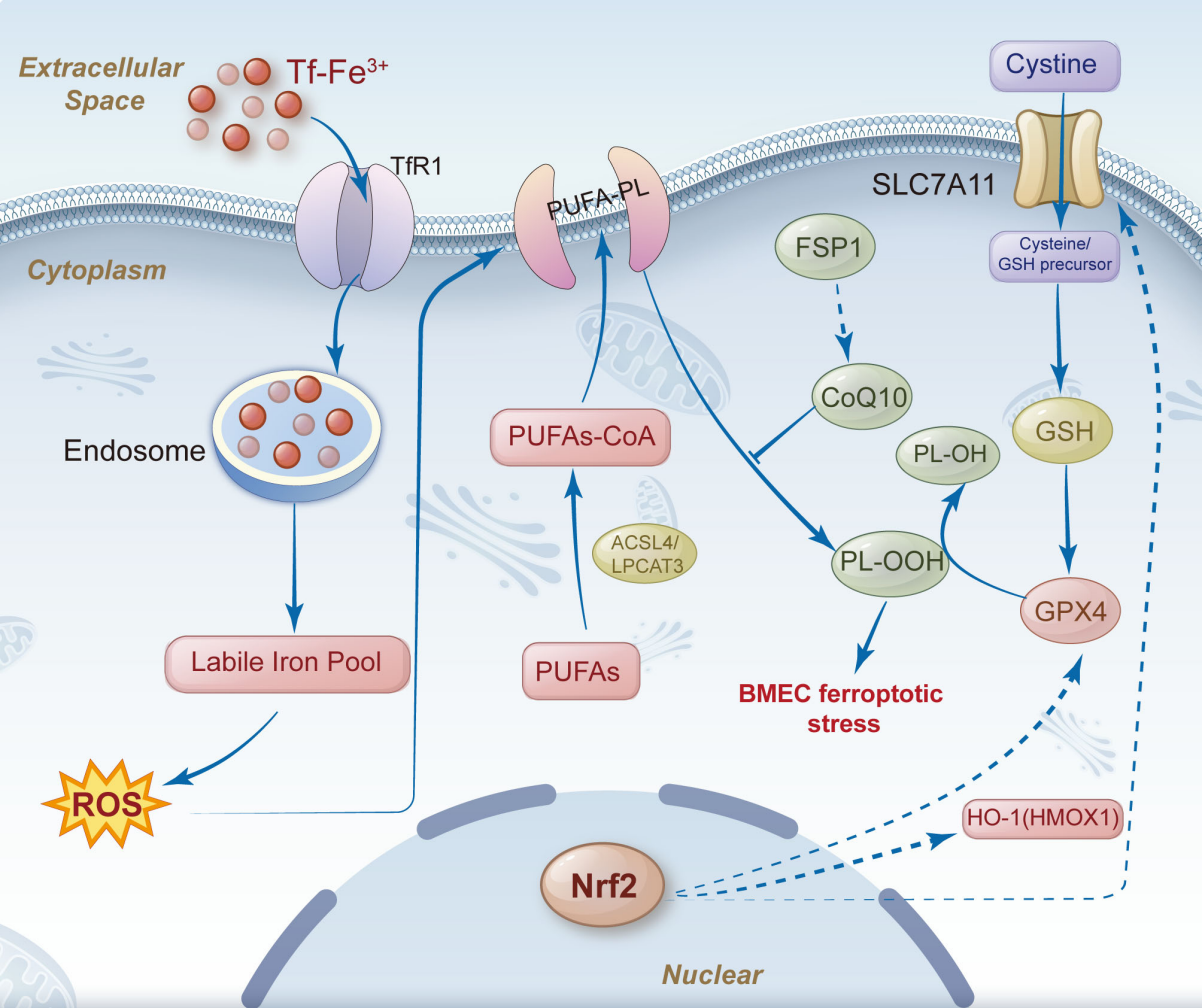
Regulatory pathways shaping BMEC susceptibility to ferroptosis. This schematic summarizes major regulatory pathways that may influence ferroptosis susceptibility in BMECs. Iron handling through transferrin receptor 1 (TfR1) and the labile iron pool can promote iron-dependent lipid peroxidation under pathological stress. Polyunsaturated fatty acid-containing phospholipids are susceptible to peroxidation, generating lipid hydroperoxides that contribute to ferroptotic injury. The SLC7A11–glutathione–GPX4 axis represents a central anti-ferroptotic defense system, whereas Nrf2-linked antioxidant signaling may support endothelial redox resilience through downstream cytoprotective programs, including HO-1 and GPX4. The FSP1–CoQ10 system is shown as a GPX4-independent ferroptosis-suppressive pathway, although its specific role in BMEC ferroptosis remains less directly established. Overall, the image illustrates a conceptual framework for BMEC ferroptosis regulation rather than a fully validated pathway in all BBB disease contexts. ACSL4, acyl-CoA synthetase long-chain family member 4; BMEC, brain microvascular endothelial cell; CoQ10, coenzyme Q10; FSP1, ferroptosis suppressor protein 1; GPX4, glutathione peroxidase 4; GSH, glutathione; HO-1, heme oxygenase-1; HMOX1, heme oxygenase 1; LPCAT3, lysophosphatidylcholine acyltransferase 3; Nrf2, nuclear factor erythroid 2-related factor 2; PL-OH, phospholipid alcohol; PL-OOH, phospholipid hydroperoxide; PUFAs, polyunsaturated fatty acids; PUFAs-CoA, polyunsaturated fatty acyl-coenzyme A species; PUFA-PL, polyunsaturated fatty acid-containing phospholipid; ROS, reactive oxygen species; SLC7A11, solute carrier family 7 member 11; Tf-Fe³^+^, transferrin-bound ferric iron; TfR1, transferrin receptor 1.

Overall, BMEC ferroptosis susceptibility appears to be shaped by the convergence of endothelial iron handling, lipid-peroxide detoxification capacity, and cerebrovascular microenvironmental stress. Iron loading and impaired GPX4/Nrf2-related defense may define the biochemical threshold for ferroptosis, whereas hypoxia, inflammatory signaling, pericyte-derived cues, and mechanical forces may determine when this threshold is crossed. Importantly, not all microenvironmental stressors should be interpreted as direct ferroptosis mechanisms; rather, they may lower BMEC resistance to ferroptotic injury in disease-specific contexts. These regulatory determinants of BMEC ferroptosis susceptibility are summarized in **Figure 1**.

## 3 Molecular mechanisms linking BMEC ferroptosis to BBB structural dysfunction

The structural barrier function of the BBB depends on the coordinated organization of tight junctions, adherens junctions, polarized transport systems, and low paracellular permeability across BMECs (3). Within this architecture, ferroptosis-associated endothelial injury may affect BBB integrity through several overlapping processes: lipid peroxidation-driven membrane stress, altered tight junction protein expression, proteolytic remodeling, abnormal junctional protein trafficking, and endothelial cell dysfunction (11, 36, 37). The available evidence is uneven across these mechanisms. Direct BMEC-specific evidence exists in selected hypoxia, ischemia, and toxicological models, whereas other pathways are supported mainly by BBB injury studies, oxidative stress literature, or non-BBB ferroptosis mechanisms (9, 11, 38). Interpreting these mechanisms according to evidence strength helps avoid treating all barrier-disruptive processes as direct consequences of BMEC ferroptosis.

### 3.1 Junctional architecture and BBB structural vulnerability

Tight junctions are the core paracellular sealing structures between adjacent BMECs. They consist of transmembrane proteins, including claudins and occludin, cytoplasmic scaffolding proteins such as ZO-1, and actin-associated regulatory elements. Among these molecules, claudin-5 is a major determinant of BBB selectivity. Mosaic deletion of claudin-5 has been shown to cause rapid non-cell-autonomous consequences of BBB leakage, supporting the importance of claudin-5 for local barrier homeostasis (39). Occludin also contributes to BBB stability, particularly under ischemic stress, where altered occludin function has been linked to BBB integrity and neurological outcome after stroke (40). ZO-1 connects transmembrane tight junction proteins to the cytoskeleton and helps preserve junctional continuity.

These junctional structures are relevant to BMEC ferroptosis because several BMEC- or BBB-focused studies have linked ferroptotic stress to tight junction remodeling (23, 41). In hypoxia-exposed bEnd.3 cells and zebrafish cerebrovascular endothelial models, ferroptosis-associated changes were accompanied by reduced CLDN5 expression and BBB disruption (9). In cerebral ischemia, solute carrier family 22 member 17 (SLC22A17) was identified as a cell death-linked regulator of tight junctions, and in human brain endothelial cultures, SLC22A17 silencing reduced TNF-α-induced ferroptosis, tight junction protein loss, and permeability changes (41). These studies provide relatively direct support for an association between endothelial ferroptotic stress and junctional remodeling. However, tight junction loss in CNS disease is not necessarily ferroptosis-dependent in all settings. It should therefore be interpreted as a structural phenotype that may accompany BMEC ferroptotic injury in specific models, rather than as a universal downstream event.

### 3.2 Lipid peroxidation-associated remodeling of junctional stability

Ferroptosis is driven by iron-dependent phospholipid peroxidation, and this process can compromise endothelial membranes and junctional organization (11, 19, 41). Reactive lipid peroxidation products, including 4-hydroxy-2-nonenal and malondialdehyde, may modify proteins, alter membrane properties, and disturb cytoskeletal–junctional coupling under oxidative stress conditions (42). In BMEC-focused hypoxia models, ferroptosis-related BBB injury was associated with increased lipid peroxidation and reduced CLDN5 expression (9). In a toxicological BMEC model, polystyrene nanoplastics induced ferroptosis-related changes in bEnd.3 cells, and ferroptosis inhibition attenuated ZO-1 reduction and BBB dysfunction (11). These findings support the concept that lipid peroxidation can be coupled to junctional instability in BMECs.

The molecular route from lipid peroxidation to tight junction remodeling remains incompletely defined. Oxidative stress may alter BBB structure through direct modification of junctional proteins, actin remodeling, mitochondrial energy stress, and transcriptional or epigenetic regulation of barrier-related genes (42). Disease-context evidence also suggests that ferroptosis-related pathways can influence BBB integrity through chromatin-associated regulatory mechanisms (43, 44). For example, in subarachnoid hemorrhage models, SIRT6 knockdown alleviated BBB disruption by inhibiting ferroptosis through SMARCA2 acetylation (45). This finding supports a broader relationship between ferroptosis, transcriptional regulation, and BBB disruption, but it does not by itself prove that lipid aldehydes directly modify claudin-5, occludin, or ZO-1 in BMECs. More precise studies using BMEC-specific lipidomics, redox proteomics, and junctional protein adduct mapping are needed to define how lipid peroxidation reshapes BBB junctions.

The key pathways linking BMEC ferroptosis to BBB dysfunction and immune–vascular signaling are summarized in **Table 1**.

**Table 1:** Key pathways linking BMEC ferroptosis to BBB dysfunction and immune–vascular signaling.

Pathway/key molecules	Relevance to BMEC ferroptosis	BBB structural role	Immune–vascular role	Evidence level/key references
Iron handling: TfR1, labile iron pool, ferritinophagy	Increased iron uptake or impaired iron handling may favor lipid peroxidation in BMECs.	May contribute to endothelial injury, CLDN5 reduction, and junctional instability in selected models.	May increase vascular oxidative stress and sensitize the BBB to inflammatory injury.	Level 1–2 (9, 12, 16);
Canonical ferroptosis defense: SLC7A11, GSH, GPX4	Loss of lipid peroxide detoxification increases ferroptosis susceptibility.	Associated with reduced barrier resistance and tight-junction protein loss in BMEC/BBB models.	Endothelial injury may facilitate downstream inflammatory amplification.	Level 1–2 (7, 9);
Nrf2-linked antioxidant defense: Nrf2, HO-1, GPX4	Supports anti-ferroptotic redox defense during endothelial injury.	BBB protection has been reported in ischemia/reperfusion and thrombolysis-related models.	May reduce ferroptosis-associated inflammatory signaling.	Level 2 (26, 28, 38);
Alternative lipid-peroxide defense: FSP1, CoQ10	GPX4-independent anti-ferroptotic pathway; BMEC-specific evidence remains limited.	Potential membrane-protective relevance, but BBB-specific validation is needed.	Immune relevance remains indirect.	Level 4 (29, 30);
Lipid peroxidation and junctional remodeling: PUFA-PLs, 4-HNE, MDA	Lipid peroxide accumulation is a core ferroptosis feature.	May disturb endothelial membranes, cytoskeletal coupling, and tight-junction organization.	Oxidized lipid mediators may influence inflammatory signaling.	Level 1–3 (11, 42);
Tight-junction regulation: CLDN5, OCLN, ZO-1, SLC22A17	Ferroptosis-associated stress has been linked to tight-junction remodeling in selected BMEC/BBB models.	CLDN5, OCLN, or ZO-1 loss or redistribution increases paracellular permeability.	Barrier leakage may expose perivascular and glial cells to inflammatory cues.	Level 1–2 (39–41);
Proteolysis and trafficking: MMP-9, Caveolin-1, p23	These pathways may interact with ferroptotic endothelial stress; direct coupling varies by model.	MMP-9 and Caveolin-1 pathways can contribute to junctional disruption; p23 stabilizes GPX4 in BMECs.	BBB leakage may amplify downstream immune activation.	Mixed Level 1–4 (23, 46, 47);
DAMP/PRR signaling: HMGB1, ATP, mtDNA, TLRs, inflammasomes	Advanced endothelial injury may release or expose danger signals.	Usually follows or accompanies endothelial injury rather than directly defining barrier structure.	May activate innate immune sensing at the BBB interface.	Level 2–3 (13, 48);
Leukocyte recruitment and NET-related injury: ICAM-1, VCAM-1, ALOX12–12-HETE, NETs	Ferroptosis-associated endothelial stress may promote a pro-inflammatory endothelial phenotype.	Leukocyte adhesion and transmigration can worsen BBB disruption.	Supports immune-cell trafficking and vascular-to-parenchymal inflammatory spread.	Level 2–4 (49–51);
Glial activation and inflammatory feedback: fibrinogen/fibrin, NLRP3, cGAS–STING, P2RX7	Inflammatory mediators may feedback to lower endothelial ferroptosis resistance.	BBB leakage allows plasma-derived signals to reach perivascular or parenchymal compartments.	Microglial and astrocytic activation may sustain inflammatory amplification.	Level 2–3 (52–54);

BBB, blood–brain barrier; BMECs, brain microvascular endothelial cells; TfR1, transferrin receptor 1; CLDN5, claudin-5; OCLN, occludin; ZO-1, zonula occludens-1; GSH, glutathione; GPX4, glutathione peroxidase 4; HO-1, heme oxygenase-1; FSP1, ferroptosis suppressor protein 1; CoQ10, coenzyme Q10; PUFA-PLs, polyunsaturated fatty acid-containing phospholipids; 4-HNE, 4-hydroxy-2-nonenal; MDA, malondialdehyde; MMP-9, matrix metalloproteinase-9; DAMPs, damage-associated molecular patterns; PRRs, pattern-recognition receptors; TLRs, Toll-like receptors; NETs, neutrophil extracellular traps.Evidence levels: Level 1, BMEC-specific direct evidence; Level 2, BBB-focused in vivo, ex vivo, or disease-model evidence; Level 3, CNS indirect or disease-context evidence; Level 4, non-CNS endothelial or general ferroptosis analogy. Mixed levels indicate that different components of the same pathway are supported by different types of evidence.

**Figure 2:**
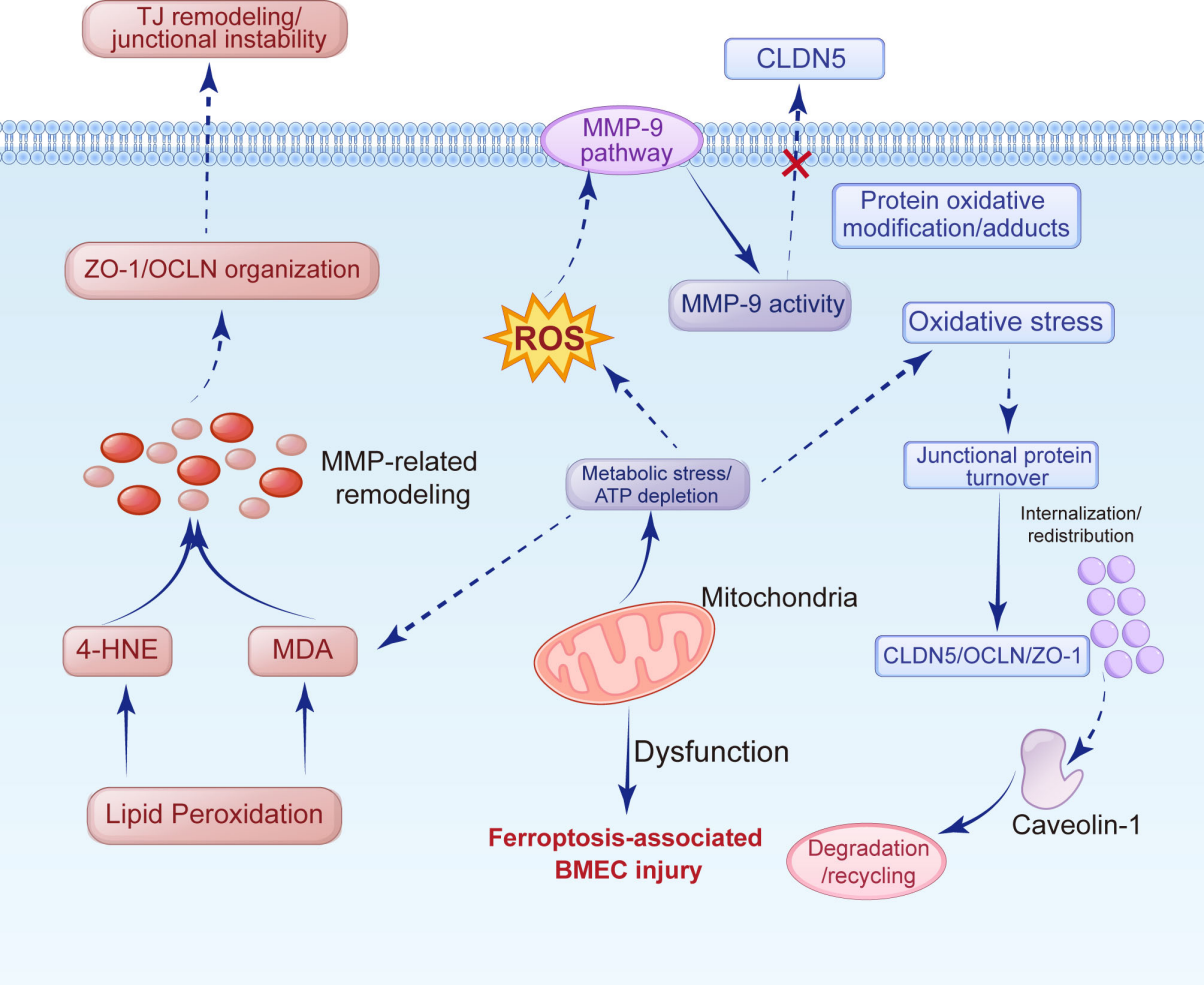
Potential mechanisms linking BMEC ferroptosis to BBB structural dysfunction. Ferroptosis-associated endothelial injury may impair BBB structure through overlapping mechanisms involving lipid peroxidation, tight junction remodeling, proteolytic activity, altered junctional protein trafficking, and endothelial metabolic stress. Lipid peroxidation products, including 4-hydroxy-2-nonenal (4-HNE) and malondialdehyde (MDA), may disturb endothelial membrane properties, cytoskeletal–junctional coupling, and tight junction organization. In selected BMEC or BBB-focused models, ferroptosis-related stress has been associated with reduced CLDN5, OCLN, or ZO-1 expression or redistribution. Proteolytic and trafficking pathways, including MMP-9-related remodeling and Caveolin-1-mediated junctional protein internalization, may further contribute to BBB disruption, although their direct coupling to BMEC ferroptosis remains incompletely defined. Arrows indicate proposed or context-dependent relationships and should not be interpreted as universal causal sequences. ATP, adenosine triphosphate; BMEC, brain microvascular endothelial cell; CLDN5, claudin-5; 4-HNE, 4-hydroxy-2-nonenal; MDA, malondialdehyde; MMP, matrix metalloproteinase; MMP-9, matrix metalloproteinase-9; OCLN, occludin; ROS, reactive oxygen species; TJ, tight junction; ZO-1, zonula occludens-1.

The proposed mechanisms by which BMEC ferroptosis may contribute to BBB structural dysfunction are illustrated in **Figure 2**.

### 3.3 Proteolytic, trafficking, and post-translational regulation of junctional proteins

Ferroptosis-associated endothelial stress may also interact with proteolytic and trafficking pathways that regulate tight junction protein stability. Matrix metalloproteinases, especially MMP-9, are established mediators of BBB injury in multiple pathological contexts because they can contribute to extracellular matrix degradation and junctional disruption. Recent work outside the BBB field suggests that MMP-9 may also modulate ferroptosis by regulating GPX4 and iron-related signaling, indicating a possible intersection between proteolysis and ferroptotic stress (46). In traumatic brain injury models, fucoxanthin attenuated BBB disruption while reducing MMP-9 expression and preserving junction-associated proteins (55). These findings support MMP-related proteolysis as a plausible amplifier of BBB structural injury. However, direct evidence that BMEC ferroptosis specifically activates MMP-dependent tight junction cleavage remains limited.

Junctional protein trafficking provides another mechanism through which BBB structure may be altered. Caveolin-1-dependent endocytosis has been implicated in the internalization and redistribution of ZO-1 and claudin-5 after ischemic injury (56). More recently, cerebral endothelial Caveolin-1-driven autophagic degradation of tight junction proteins was linked to post-stroke BBB disruption, and small extracellular vesicle treatment was reported to antagonize this process (47). These trafficking pathways are highly relevant to BBB dysfunction, but they should be distinguished from ferroptosis-specific mechanisms unless ferroptosis readouts are measured in the same model. More direct BMEC-focused evidence comes from studies of p23, which was reported to protect against cerebral ischemia/reperfusion-induced BBB injury by inhibiting BMEC ferroptosis and stabilizing GPX4 (23). Together, these observations suggest that ferroptosis-associated BBB structural dysfunction may involve not only lipid peroxidation, but also altered protein stability, chaperone function, and junctional protein trafficking. The relative contribution of each pathway is likely model-dependent.

### 3.4 Assessing ferroptosis-associated BBB structural dysfunction

Because many injury pathways converge on BBB leakage, ferroptosis-associated BBB dysfunction should be evaluated using both ferroptosis-specific and barrier-specific readouts. Ferroptosis readouts may include iron accumulation, lipid reactive oxygen species, malondialdehyde or 4-hydroxy-2-nonenal levels, GPX4 or SLC7A11 downregulation, mitochondrial morphological changes, and rescue by ferroptosis inhibitors (57–59). Barrier readouts should include tight junction protein expression and localization, transendothelial electrical resistance, paracellular tracer permeability, Evans blue or dextran leakage, and ultrastructural examination of endothelial junctions (60–62). Studies that combine ferroptosis markers, BBB functional assays, and rescue experiments provide stronger support than studies measuring either ferroptosis markers or BBB leakage alone (11, 23).

Advanced imaging methods can further quantify BBB permeability *in vivo*, but they do not by themselves identify ferroptosis as the causal mechanism. Quantitative positron emission tomography (PET) imaging and kinetic modeling have been developed to measure molecular BBB permeability in humans (63). Tracer kinetic modeling based on dynamic contrast-enhanced imaging has also been used to detect heterogeneous BBB permeability in Alzheimer’s disease and dementia with Lewy bodies (64). These approaches are valuable for assessing barrier dysfunction at the tissue or clinical level, but they need to be paired with cell-type-specific molecular evidence before being interpreted as indicators of BMEC ferroptosis. The strongest future studies will therefore combine BMEC-specific ferroptosis markers, spatially resolved BBB permeability assays, immune or vascular readouts, and functional rescue experiments to determine whether endothelial ferroptosis acts as a driver, amplifier, or consequence of BBB structural dysfunction.

Taken together, BMEC ferroptosis-associated BBB dysfunction should be interpreted as a composite phenotype rather than a single molecular event. Lipid peroxidation, tight junction remodeling, proteolytic activity, altered trafficking, and endothelial metabolic stress may converge on barrier leakage, but their causal hierarchy remains model-dependent. Therefore, future studies should combine BMEC-specific ferroptosis markers, barrier-function assays, endothelial localization, immune or vascular readouts, and rescue experiments before attributing BBB disruption directly to BMEC ferroptosis.

## 4 Immune–vascular cascade from endothelial ferroptosis to neuroinflammation

The immunological relevance of BMEC ferroptosis lies not only in its potential contribution to BBB structural dysfunction, but also in its capacity to reshape immune signaling at the neurovascular interface (65–67). When endothelial redox imbalance, lipid peroxidation, and junctional remodeling occur together, vascular injury may be translated into a sequence of inflammatory events involving danger-signal release, innate immune sensing, leukocyte recruitment, glial activation, and feedback amplification (68–70). However, this cascade should not be interpreted as a uniformly proven linear pathway. Some steps are supported by BMEC- or BBB-focused studies, whereas others are inferred from ischemic stroke, sepsis, neurodegeneration, non-CNS endothelial systems, or broader innate immune biology. This section therefore frames endothelial ferroptosis as a plausible immune–vascular amplifier of neuroinflammation while preserving the distinction between direct evidence and mechanistic inference.

### 4.1 Ferroptosis-associated endothelial danger signals and innate immune sensing

Advanced ferroptotic injury can be accompanied by membrane damage and release or exposure of DAMPs (71–73). In the BBB context, this process is particularly important because BMECs lie at the boundary between the circulation, perivascular compartment, and brain parenchyma. HMGB1 is one of the most frequently discussed DAMPs in this setting. Endothelial N-methyl-D-aspartate receptor activation has been reported to regulate endothelial ferroptosis through the PP2A–AMPK–HMGB1 axis, suggesting a potential route by which ferroptotic endothelial stress may intersect with HMGB1-associated inflammatory signaling (48). In ischemic stroke and hemorrhagic transformation, extracellular HMGB1 has also been discussed as a marker and mediator of BBB disruption, edema, and inflammatory amplification (74).

At the BBB interface, DAMPs may engage pattern-recognition receptors (PRRs) expressed by endothelial cells, perivascular immune cells, microglia, astrocytes, and other neurovascular-unit components (13, 75, 76). Toll-like receptors and inflammasomes are particularly relevant because they provide a mechanism through which vascular danger signals can be converted into innate immune activation at the barrier (13). For example, the HMGB1/TLR4/NF-κB network has been implicated in cerebral ischemia/reperfusion-induced neuroinflammation, although this evidence does not by itself prove that BMEC-derived HMGB1 is the initiating event (77). Thus, HMGB1-related signaling is best interpreted as a mechanistically plausible bridge between endothelial injury and inflammatory activation, rather than as a fully established BMEC-specific causal pathway.

Ferroptosis-associated lipid mediators may provide an additional danger-signal layer. In a non-CNS ischemia/reperfusion model, inhibition of the ALOX12–12-HETE axis reduced endothelial ferroptosis-mediated neutrophil extracellular trap formation, suggesting that lipid peroxidation products can promote pathological endothelial–neutrophil interactions (51). Because this evidence comes from the lung rather than the BBB, it should be used as a mechanistic analogy, not as direct evidence for BMECs. Nevertheless, it supports the broader concept that lipid peroxidation products generated during endothelial ferroptotic stress may shape inflammatory cell behavior.

### 4.2 Plasma-derived danger signals and endothelial–immune cell trafficking

If BBB integrity is compromised in settings where endothelial ferroptosis is present, blood-derived proteins may enter the perivascular space and brain parenchyma (5, 78, 79). These extravasated molecules can act as exogenous danger signals that amplify local neuroinflammation (80). Multi-omic profiling has shown that blood exposure can induce disease-relevant microglial programs in neurodegenerative contexts (81). Fibrinogen and fibrin are especially important in this regard (82, 83). Fibrinogen has been reported to prime the microglial NLRP3 inflammasome and promote pro-inflammatory signaling through extracellular vesicle release, supporting the idea that plasma-derived proteins can convert vascular leakage into glial inflammatory activation (52).

Endothelial activation also provides the structural and molecular basis for leukocyte recruitment (5, 84). Under neuroinflammatory conditions, immune-cell trafficking across the BBB proceeds through coordinated steps, including leukocyte capture, adhesion, crawling, diapedesis, and post-barrier migration (49). Ferroptosis-associated endothelial stress may intersect with this process by increasing oxidative stress, modifying endothelial membrane lipids, and promoting a pro-inflammatory endothelial phenotype. However, direct evidence that BMEC ferroptosis itself is sufficient to induce ICAM-1, VCAM-1, or other adhesion molecules remains limited and model-dependent. Therefore, adhesion molecule upregulation should be framed as a potential consequence of ferroptosis-associated endothelial activation rather than as an established universal feature of BMEC ferroptosis.

Neutrophils may be among the earliest peripheral immune cells recruited after BBB injury. NET formation can further injure endothelial barriers and promote inflammatory amplification (50, 85). In sepsis-associated encephalopathy, inhibition of neutrophil extracellular traps alleviated BBB disruption and cognitive dysfunction through Wnt3/β-catenin/TCF4 signaling, supporting a role for NETs in barrier injury and neuroinflammatory propagation (50). Together with the ALOX12–12-HETE evidence from non-CNS endothelium, these findings suggest that ferroptosis-linked lipid signals and NET-associated endothelial injury may converge on immune-cell trafficking pathways. The direct contribution of BMEC ferroptosis to NET formation at the BBB, however, still requires experimental validation.

### 4.3 Conversion of vascular-derived signals into glial inflammatory activation

Once vascular-derived danger signals cross or accumulate near the BBB, they may be sensed by microglia and astrocytes (79, 81, 86). Microglia are particularly responsive to blood-derived molecules, DAMPs, cytokines, and extracellular vesicles, and can convert these signals into inflammatory transcriptional programs (81, 87, 88). Fibrinogen-induced NLRP3 activation provides one example of how BBB leakage can be translated into microglial inflammasome activity (52). In ischemia/reperfusion injury, ferritinophagy-associated microglial ferroptosis has also been linked to neuroinflammation through activation of the cGAS–STING pathway (53). These findings support a vascular-to-glial inflammatory conversion model, although they do not prove that BMEC ferroptosis is the sole or primary upstream trigger.

Astrocytes may also participate in this conversion process. As endfeet-associated components of the BBB, astrocytes respond to endothelial injury, plasma protein leakage, and inflammatory mediators. In acute injury, astrocyte-derived signals may contribute to barrier stabilization or repair, whereas persistent exposure to vascular danger signals can promote reactive astrocyte states and inflammatory amplification (89–91). Because astrocyte responses are stage- and context-dependent, they should not be described simply as protective or damaging. In the context of endothelial ferroptosis, the most cautious interpretation is that astrocytes may help transmit, buffer, or amplify vascular-derived inflammatory signals depending on injury timing and disease context.

The resulting glial activation can reshape the neurovascular microenvironment through cytokine production, inflammasome signaling, extracellular vesicle release, and altered redox metabolism (52, 81, 92). This is the point at which endothelial ferroptosis becomes immunologically important: it may provide a vascular source of danger signals and barrier dysfunction that allows peripheral and perivascular inflammatory cues to engage glial immune programs. However, the evidence is currently strongest for individual components of this pathway—BBB leakage, HMGB1/TLR4/NF-κB signaling, fibrinogen–NLRP3 activation, NET-associated barrier injury, and microglial cGAS–STING signaling—rather than for a single fully validated BMEC ferroptosis-to-glial activation axis (10, 50, 93).

### 4.4 Inflammatory feedback that lowers endothelial ferroptosis resistance

Endothelial ferroptosis and neuroinflammation are unlikely to operate as a one-way sequence. Instead, inflammatory signaling may feed back onto BMECs and lower their resistance to ferroptotic stress (41, 66). Pro-inflammatory mediators can alter endothelial redox balance, suppress antioxidant defenses, increase iron-related stress, and promote lipid peroxidation (41, 94, 95). In vascular endothelial cells, lipopolysaccharide (LPS)-induced inflammation has been linked to endothelial ferroptosis, and Nrf2-mediated redox balance was reported to alleviate this process (94). Although this finding is not BMEC-specific, it supports the broader principle that inflammatory stress can interact with endothelial ferroptosis pathways.

Several molecular nodes may help sustain this feedback loop. P2RX7, a purinergic receptor responsive to extracellular ATP, has been implicated in endothelial ferroptosis and hemorrhagic transformation after ischemic injury; P2RX7 blockade attenuated endothelial ferroptosis by inhibiting ERK1/2 and p53 signaling (54). Nrf2 represents another key node because it links oxidative stress, ferroptosis resistance, inflammation, and BBB protection (26, 38). In cerebral ischemic stroke, Nrf2 activation ameliorated BBB injury by regulating ferroptosis and inflammation, supporting the relevance of antioxidant defense pathways in interrupting endothelial injury–inflammation coupling (38).

These feedback mechanisms may help explain how an acute vascular insult becomes prolonged neuroinflammatory injury. Endothelial ferroptosis-associated BBB dysfunction may facilitate DAMP exposure, plasma protein leakage, immune-cell trafficking, and glial activation; in turn, inflammatory mediators produced by immune and glial cells may further weaken endothelial antioxidant defenses and enhance ferroptosis susceptibility (28, 41, 52). This loop remains incompletely mapped at the BMEC-specific level, but it provides a useful immune–vascular framework for interpreting disease progression. Future studies should test this model using BMEC-selective ferroptosis markers, barrier-function readouts, immune-cell profiling, and temporal rescue experiments that determine whether endothelial ferroptosis acts as an initiating trigger, an amplifier, or a consequence of neuroinflammatory signaling.

**Figure 3:**
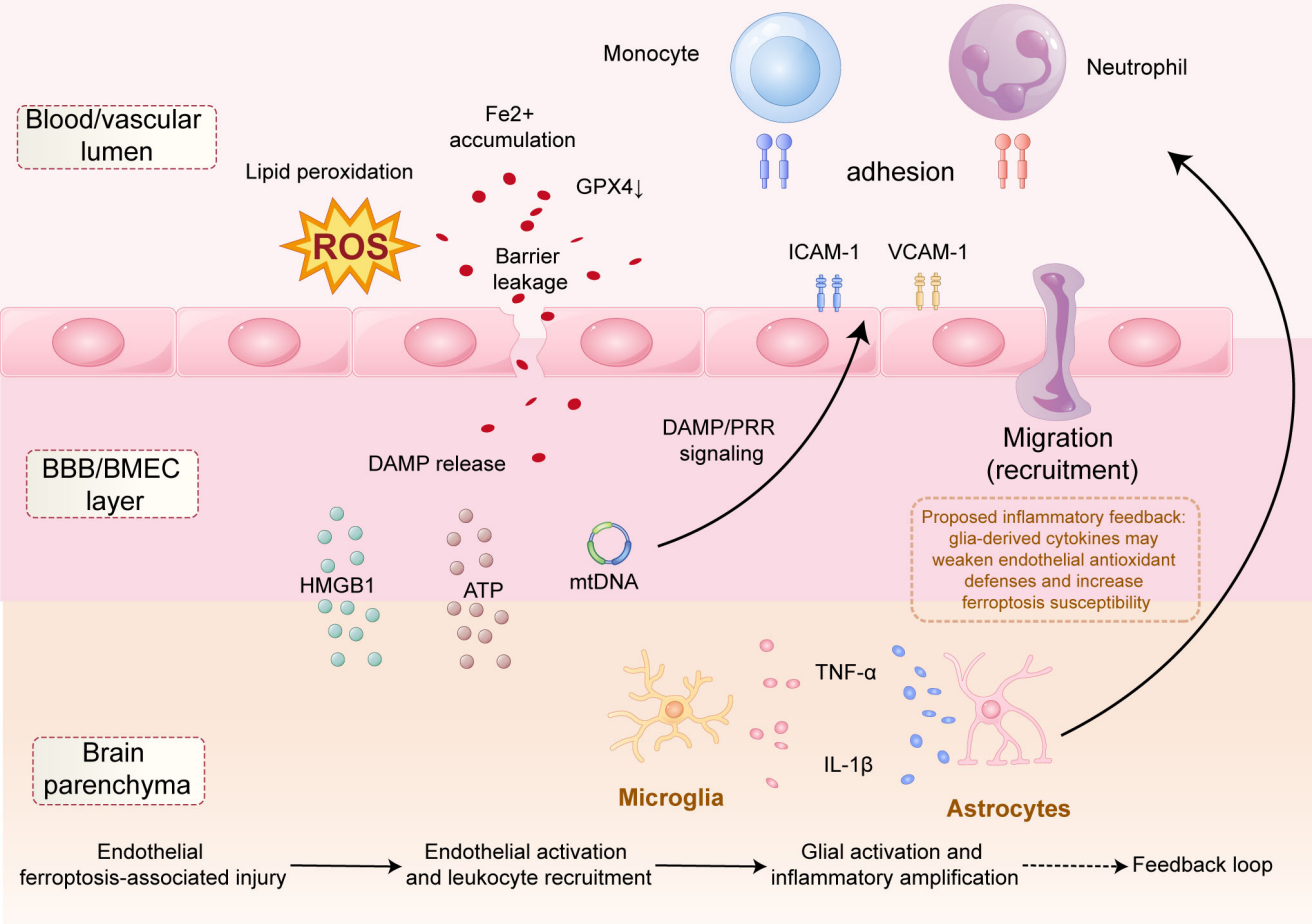
Immune–vascular cascade from endothelial ferroptosis-associated injury to neuroinflammation. This schematic illustrates a proposed immune–vascular cascade through which endothelial ferroptosis-associated injury may contribute to BBB dysfunction and neuroinflammatory amplification. At the BBB/BMEC layer, ferroptosis-related stress is represented by lipid peroxidation, ROS generation, Fe²^+^ accumulation, GPX4 reduction, and barrier leakage. These endothelial changes may promote the release or exposure of representative danger signals, including HMGB1, ATP, and mitochondrial DNA, which can engage DAMP/PRR signaling at the neurovascular interface. Endothelial activation is further illustrated by ICAM-1 and VCAM-1 expression, leukocyte adhesion, and recruitment or migration of monocytes and neutrophils across the injured barrier. In the brain parenchyma, microglia and astrocytes may convert vascular-derived danger signals into inflammatory cytokine production, including TNF-α and IL-1β, thereby contributing to inflammatory amplification. The feedback arrow indicates that glia-derived cytokines and inflammatory mediators may further weaken endothelial antioxidant defenses and increase BMEC ferroptosis susceptibility. The cascade is presented as a conceptual and evidence-stratified model rather than a fully established linear pathway; direct BMEC-specific evidence is strongest for selected upstream endothelial ferroptosis events, whereas several downstream immune and glial links remain context-dependent or inferential. ATP, adenosine triphosphate; BBB, blood–brain barrier; BMEC, brain microvascular endothelial cell; DAMP, damage-associated molecular pattern; Fe²^+^, ferrous iron; GPX4, glutathione peroxidase 4; HMGB1, high-mobility group box 1; ICAM-1, intercellular adhesion molecule 1; IL-1β, interleukin-1β; mtDNA, mitochondrial DNA; PRR, pattern-recognition receptor; ROS, reactive oxygen species; TNF-α, tumor necrosis factor-α; VCAM-1, vascular cell adhesion molecule 1.

Taken together, this immune–vascular cascade should be viewed as a bidirectional amplification model rather than a fully established linear pathway. The strongest evidence currently supports endothelial ferroptotic stress, BBB leakage, and selected inflammatory readouts in acute vascular injury models, whereas DAMP/PRR signaling, leukocyte recruitment, NET formation, and glial activation remain more context-dependent or inferential in relation to BMEC ferroptosis. Thus, the major immunological implication of BMEC ferroptosis is not that it alone initiates neuroinflammation, but that it may lower the threshold for vascular-to-parenchymal inflammatory propagation. This bidirectional immune–vascular amplification model is illustrated in **Figure 3**.

## 5 Disease-specific evidence linking endothelial ferroptosis to BBB dysfunction and neuroinflammation

The contribution of endothelial ferroptosis to BBB dysfunction differs across neurological diseases in both evidence strength and temporal pattern. Acute vascular insults, such as ischemic stroke and reperfusion injury, provide relatively direct evidence because hypoxia, oxidative stress, BBB leakage, ferroptosis markers, and endothelial injury can be examined within defined experimental windows (9, 23, 26, 38). By contrast, chronic neurodegenerative diseases such as Alzheimer’s disease (AD) and Parkinson’s disease (PD) involve prolonged interactions among iron dyshomeostasis, protein aggregation, vascular remodeling, immune activation, and impaired clearance (96–98). In these settings, endothelial ferroptosis may act as a vascular amplifier of disease progression, but BMEC-specific causality is often less established. This section therefore compares disease contexts according to the strength of BMEC-specific evidence, BBB-focused evidence, and indirect neurovascular evidence.

### 5.1 Ischemic stroke and reperfusion injury: comparatively strong BMEC/BBB evidence

Ischemic stroke and reperfusion injury currently provide some of the strongest disease-context evidence linking BMEC ferroptosis to BBB dysfunction (23, 28, 99). Hypoxia and subsequent reoxygenation impose metabolic stress, iron-dependent oxidative injury, and lipid peroxidation on brain endothelial cells (23, 26). In hypoxia-exposed bEnd.3 cells and zebrafish cerebrovascular endothelial models, ferroptosis-associated changes were accompanied by CLDN5 downregulation and BBB disruption, providing direct support for a link between BMEC ferroptosis and barrier injury (9). This evidence makes ischemic stroke a useful anchor disease for evaluating endothelial ferroptosis at the BBB.

Several post-ischemic and thrombolysis-related models further support this association. Lipocalin-2 was reported to aggravate BBB dysfunction after intravenous thrombolysis by promoting endothelial cell ferroptosis through the HMGB1/Nrf2/HO-1 pathway (28). Nrf2-linked antioxidant defense also appears relevant in ischemia/reperfusion injury: edaravone dexborneol protected against cerebral ischemia/reperfusion-induced BBB damage by inhibiting ferroptosis through Nrf2/HO-1/GPX4 signaling, and Nrf2 activation was reported to ameliorate BBB injury after cerebral ischemic stroke by regulating ferroptosis and inflammation (26, 38). These studies support the concept that endothelial redox defense capacity influences post-stroke BBB vulnerability.

Additional pathways indicate that endothelial ferroptosis may be involved not only in early BBB leakage, but also in hemorrhagic transformation and immune–vascular amplification (28, 54). P2RX7 blockade attenuated endothelial ferroptosis and reduced hyperglycemia-associated hemorrhagic transformation after middle cerebral artery occlusion by inhibiting ERK1/2 and p53 signaling (54). Targeted delivery of a ferroptosis inhibitor was also reported to reduce recombinant tissue plasminogen activator-induced hemorrhagic transformation, suggesting that ferroptosis-directed strategies may protect the BBB in selected reperfusion settings (100). Macrophage-derived exosomal THBS1 has been shown to exacerbate cerebral ischemia–reperfusion injury by inducing ferroptosis in endothelial cells, further connecting immune-cell communication with endothelial ferroptotic injury (101). Together, these data support ischemic stroke as the disease setting in which endothelial ferroptosis has the most coherent BBB-related evidence. However, even here, endothelial ferroptosis should be interpreted as one contributor to BBB disruption and inflammatory amplification, rather than the sole mechanism of post-stroke vascular injury.

### 5.2 Alzheimer’s disease and cerebral amyloid angiopathy: emerging vascular evidence

In AD, the relationship between endothelial ferroptosis and BBB dysfunction appears more chronic and multifactorial (96, 97, 102). Iron dyshomeostasis, amyloid-β accumulation, oxidative stress, impaired vascular clearance, and microvascular remodeling all contribute to the neurovascular environment in which ferroptosis may develop (8, 102, 103). Unlike ischemic stroke, however, AD does not usually involve a single acute vascular insult. Instead, endothelial ferroptosis is more plausibly viewed as an emerging mechanism that may amplify chronic BBB dysfunction and neuroinflammation (104).

Several studies support a vascular link between amyloid pathology, ferroptosis, and BBB disruption. β-amyloid protein has been reported to induce mitophagy-dependent ferroptosis through the CD36/PINK1/PARKIN pathway, leading to BBB destruction in AD-related models (105). More recent brain endothelial evidence suggests that amyloid-β aggregates can induce vasculopathy through ferroptosis in brain endothelial cells (106). Postmortem evidence also supports the vascular relevance of this pathway: structural changes in cerebral microvasculature induced by ferroptosis have been reported to contribute to BBB disruption in AD autopsy samples (107). These findings strengthen the rationale for considering endothelial ferroptosis in AD, but postmortem and disease-context studies cannot fully establish temporal causality.

CAA and chronic hypoperfusion may further increase endothelial ferroptosis susceptibility (22, 106). In hypoperfusion-induced white matter injury, myelin debris endocytosis by brain endothelial cells caused endothelial iron overload and disrupted iron availability for oligodendroglial lineage cells, providing a mechanism by which chronic vascular stress may reshape endothelial iron handling (22). In AD and CAA, vascular amyloid deposition, impaired clearance, and chronic perfusion deficits may therefore create a pro-ferroptotic endothelial environment (108, 109). Nevertheless, direct evidence that BMEC ferroptosis initiates BBB failure or neuroinflammatory conversion in AD/CAA remains less established than in acute ischemic models, where endothelial ferroptosis, tight-junction loss, permeability changes, and rescue interventions have been examined within temporally defined injury windows (23, 41). The most balanced interpretation is that endothelial ferroptosis may serve as a vascular amplifier in AD/CAA, linking amyloid-associated vascular injury, BBB leakage, iron dyshomeostasis, and inflammatory signaling (22, 106).

### 5.3 Parkinson’s disease: mainly indirect neurovascular and inflammatory evidence

Compared with ischemic stroke and AD/CAA, evidence for BMEC ferroptosis in PD is more indirect. Ferroptosis in PD has been studied mainly in relation to neuronal iron accumulation, α-synuclein pathology, oxidative stress, and dopaminergic vulnerability (110–112). Human α-synuclein aggregation has been reported to activate ferroptosis in neuronal populations, and LRRK2 has been linked to α-synuclein-induced neuroinflammation and ferroptosis through the p62–Keap1–Nrf2 pathway (113, 114). These findings support ferroptosis as a relevant mechanism in PD biology, but they do not directly demonstrate BMEC ferroptosis.

The vascular and immune evidence in PD is nevertheless relevant to the broader immune–vascular framework. BBB alterations have increasingly been discussed in PD pathogenesis and therapy, and neurovascular dysfunction may contribute to impaired immune regulation, altered transport, and vulnerability of the substantia nigra microenvironment (115). Endothelial and perivascular inflammatory changes have also been reported. For example, decreased neuronal and increased endothelial fractalkine expression have been associated with neuroinflammation in PD and related disorders (116). Enlarged perivascular spaces have been linked to peripheral inflammation, disease progression, and motor symptoms in PD, suggesting that perivascular remodeling may be part of the inflammatory disease environment (117). In addition, a brain-chip model of PD showed that inflammatory astrocytes can contribute to BBB impairment, reinforcing the idea that glial–vascular interactions are important in PD-related barrier dysfunction (118).

These observations support the inclusion of PD as a disease context in which ferroptosis, BBB dysfunction, and neuroinflammation may intersect. However, the direct BMEC ferroptosis evidence remains limited. Therefore, PD should not be presented as equivalent to ischemic stroke in evidentiary strength. A more appropriate conclusion is that PD provides indirect neurovascular and inflammatory evidence consistent with the immune–vascular framework, while requiring future studies that directly test whether BMEC ferroptosis occurs in substantia nigra microvascular compartments and whether it contributes to immune-cell trafficking or glial activation.

### 5.4 Cross-disease comparison and evidence strength

Across neurological diseases, several convergent patterns can be identified. Iron dyshomeostasis, impaired GPX4/SLC7A11-dependent defense, lipid peroxidation, junctional remodeling, and inflammatory signaling recur across ischemic, neurodegenerative, and inflammatory contexts (81, 106, 110). Nrf2-related antioxidant responses appear repeatedly as protective nodes in BBB-focused ferroptosis studies, especially in ischemic stroke models (26, 28, 38). DAMP release, HMGB1-related signaling, plasma protein leakage, and glial inflammatory activation may connect vascular injury to the immune–vascular cascade described above (52, 93).

At the same time, the upstream triggers and evidentiary strength differ substantially. In ischemic stroke and reperfusion injury, endothelial ferroptosis is temporally linked to hypoxia/reoxygenation, thrombolysis-associated BBB injury, hemorrhagic transformation, and early inflammatory amplification (9, 93). In AD/CAA, endothelial ferroptosis is better interpreted as an emerging chronic vascular mechanism associated with amyloid toxicity, microvascular remodeling, hypoperfusion, and impaired iron handling (102, 106). In PD, ferroptosis evidence is stronger in neurons and disease-related inflammatory pathways than in BMECs, while vascular and perivascular studies suggest a plausible but still indirect connection to BBB dysfunction (110, 119).

Overall, the disease-specific evidence should therefore be interpreted in a graded manner. Ischemic stroke and reperfusion injury provide the clearest temporal and mechanistic links among BMEC ferroptosis, tight-junction loss, BBB leakage, and inflammatory amplification. AD/CAA offers emerging vascular evidence in which endothelial ferroptosis may act as a chronic amplifier of amyloid-associated microvascular injury, whereas PD remains mainly hypothesis-generating from a BMEC perspective because ferroptosis evidence is stronger in neurons and inflammatory pathways than in brain endothelial compartments. Presenting the diseases in this order helps avoid treating all disease settings as equally established and clarifies where future BMEC-specific studies are most needed. The disease-specific evidence strength and therapeutic implications of endothelial ferroptosis at the BBB are summarized in **Table 2**.

**Table 2:** Disease-specific evidence strength and therapeutic implications of endothelial ferroptosis at the BBB.

Disease context	Disease-specific context	BMEC/BBB evidence	Immune–vascular cascade role	Evidence strength category and therapeutic implications	Key references
Ischemic stroke/reperfusion injury	Hypoxia–reoxygenation; thrombolysis-associated BBB injury; hemorrhagic transformation risk; hyperglycemic stress	BMEC hypoxia models link ferroptosis to CLDN5 loss and BBB disruption; LCN2, Nrf2/HO-1/GPX4, and P2RX7, p23, and targeted-delivery studies implicate endothelial ferroptosis in post-ischemic BBB injury	Acute endothelial injury may promote BBB leakage, DAMP exposure, immune-cell recruitment, and inflammatory feedback	Relatively strong BMEC/BBB-focused preclinical evidence: Nrf2/GPX4 support, P2RX7 inhibition, and targeted ferroptosis-inhibitor delivery are plausible strategies, but BMEC-specific clinical validation is lacking.	(23, 26, 28, 54, 100)
Alzheimer’s disease/cerebral amyloid angiopathy	Aβ-associated vascular toxicity; CAA; chronic hypoperfusion; impaired endothelial/pericyte iron handling	Aβ-related BBB damage has been linked to ferroptosis in pericytes and brain endothelial cells; AD autopsy and chronic hypoperfusion studies support microvascular ferroptosis or endothelial iron-loading associations	Chronic BBB leakage and vascular amyloid injury may expose glia to plasma-derived and endothelial danger signals, supporting low-grade inflammatory amplification	Emerging vascular/disease-context evidence: Iron-handling modulation, Nrf2/GPX4 support, and vascular protection remain hypothesis-generating; temporal BMEC-specific causality remains incompletely established.	(22, 105–107)
Parkinson’s disease	Nigral iron dyshomeostasis; α-synuclein aggregation; LRRK2-related microglial ferroptosis/inflammation; perivascular remodeling	Ferroptosis evidence is stronger in neurons and microglia than in BMECs; PD studies support BBB alterations, endothelial inflammatory changes, perivascular-space remodeling, and astrocyte–vascular BBB impairment	Perivascular inflammation and glial–vascular dysfunction may contribute to BBB impairment and inflammatory propagation	Mainly indirect neurovascular evidence: Endothelial-targeted anti-ferroptosis therapy remains speculative until direct evidence of BMEC ferroptosis in PD microvascular compartments is available.	(113–118)

AD, Alzheimer’s disease; Aβ, amyloid-β; BBB, blood–brain barrier; BMECs, brain microvascular endothelial cells; CAA, cerebral amyloid angiopathy; CLDN5, claudin-5; DAMPs, damage-associated molecular patterns; GPX4, glutathione peroxidase 4; HO-1, heme oxygenase-1; HT, hemorrhagic transformation; LCN2, lipocalin-2; LRRK2, leucine-rich repeat kinase 2; Nrf2, nuclear factor erythroid 2-related factor 2; P2RX7, purinergic receptor P2X7; PD, Parkinson’s disease. Evidence strength categories: “Relatively strong BMEC/BBB-focused preclinical evidence” indicates studies with BMEC or BBB functional readouts together with ferroptosis-related markers. “Emerging vascular/disease-context evidence” indicates vascular or BBB-associated findings in disease models or human tissue without fully resolved BMEC-specific causality. “Mainly indirect neurovascular evidence” indicates that ferroptosis evidence is stronger in non-endothelial CNS compartments or inflammatory disease contexts than in BMECs.

## 6 Therapeutic strategies, BMEC-specific targeting, and translational barriers for targeting endothelial ferroptosis at the BBB

Targeting endothelial ferroptosis represents a promising therapeutic concept for preserving BBB integrity, but the current evidence remains predominantly preclinical (10, 11). Therapeutic strategies should therefore be evaluated according to the strength of BMEC specificity, BBB functional readouts, ferroptosis markers, immune or inflammatory outcomes, delivery feasibility and potential systemic risks (12, 38). Interventions with relatively direct BBB- or BMEC-focused evidence include iron chelation (120), modulation of iron uptake (12), enhancement of GPX4- or Nrf2-associated antioxidant defenses (23, 38), and delivery of ferroptosis inhibitors to injured cerebrovascular endothelium (12). Other approaches, including repurposed metabolic drugs, sigma-1 receptor agonists, mevalonate pathway modulation, extracellular vesicles, and endothelial-targeted nanomedicine, remain promising but require more rigorous validation in BMEC-specific and human BBB models. A clinically useful framework should also consider how therapies can be directed to BMECs, how treatment responses can be monitored with biomarkers, and how broad or prolonged ferroptosis blockade may affect physiological immune, redox, and repair functions.

### 6.1 Iron-handling and antioxidant interventions with BBB-focused evidence

Iron chelation is one of the most direct ways to reduce the iron-dependent lipid peroxidation that drives ferroptosis. Deferoxamine (DFO), a clinically used iron chelator, has been reported to reduce endothelial ferroptosis and preserve cerebrovascular function after experimental traumatic brain injury (120). This provides relatively strong preclinical support for iron chelation as a BBB-protective strategy. However, DFO has pharmacokinetic and BBB-delivery limitations, and its effects are not necessarily restricted to BMECs. Nanotechnology-based DFO delivery systems, including carrier-free DFO nanoparticles and mesoporous silica nanoplatforms derived from DFO analogues, have been developed to improve brain delivery and neuroprotective chelation capacity (121). These platforms are relevant to endothelial ferroptosis therapy, but most remain preclinical delivery solutions rather than clinically validated BBB-targeted treatments.

A related strategy is to limit pathological iron uptake at the BBB. TfR1-mediated iron handling is especially relevant because BMECs participate in iron exchange between the circulation and the CNS. Rosmarinic acid-loaded liposomes were reported to suppress ferroptosis in ischemic brain by inhibiting TfR1 in BMECs, providing one of the more BMEC-focused examples of iron-handling intervention (12). This type of evidence supports the idea that therapeutic modulation of endothelial iron uptake may protect BBB function, but it should be distinguished from more general iron chelation strategies.

Enhancement of GPX4- and Nrf2-associated antioxidant defenses provides another major therapeutic route. Se-(methyl)-selenocysteine was reported to ameliorate BBB disruption after focal cerebral ischemia through ferroptosis inhibition and tight junction upregulation in an Akt/GSK3β-dependent manner (122). Selenium-based nanoparticles have also shown anti-ferroptotic effects in hemorrhagic brain injury models through Nrf2/GPX4-related mechanisms (123). In cerebral ischemia/reperfusion injury, edaravone dexborneol protected the BBB by inhibiting ferroptosis through activation of the Nrf2/HO-1/GPX4 pathway (26). Nrf2 activation has also been linked to BBB protection after ischemic stroke through regulation of ferroptosis and inflammation (38). These studies support Nrf2–GPX4 signaling as a recurring protective axis, but the contribution of BMEC-autonomous antioxidant signaling needs to be separated from neuronal, glial, and systemic anti-inflammatory effects.

Overall, iron chelation, TfR1 modulation, selenium-related GPX4 support, and Nrf2 activation represent the most coherent preclinical therapeutic themes. Nevertheless, they should not be presented as established clinical strategies for endothelial ferroptosis at the BBB. The strongest evidence comes from models that combine ferroptosis readouts, BBB permeability or tight junction readouts, and functional rescue experiments; studies lacking one of these elements should be interpreted more cautiously.

### 6.2 Repurposed and pathway-based strategies: promising but indirect evidence

Drug repurposing may accelerate therapeutic exploration, but many repurposed agents have only indirect evidence for BMEC ferroptosis (124–126). Metformin is one example. It has been discussed as a neurovascular-unit protective agent in CNS disorders, and *in vitro* BBB models indicate that metformin can cross the BBB through organic cation transporters, including Oct1 (127, 128). Metformin has also been reported to inhibit ferroptosis through AMPK activation after spinal cord injury (129). These findings support metformin as a mechanistically plausible candidate, but they do not yet establish that metformin directly suppresses BMEC ferroptosis to restore BBB integrity in neurological disease.

Dimethyl fumarate (DMF) and other Nrf2-related agents also require careful framing. DMF can activate antioxidant signaling and has been linked to ferroptosis suppression in several injury contexts, including brain injury and chronic hypoperfusion models (130–132). However, the available evidence often involves neuronal, glial, renal, or systemic oxidative stress pathways rather than BMEC-specific ferroptosis (131–133). Therefore, DMF is best discussed as a pathway-based Nrf2 modulator with potential relevance to BBB protection, not as a validated endothelial ferroptosis therapy.

Other repurposed or pathway-based approaches are even more indirect. The mevalonate pathway can influence ferroptosis resistance through CoQ10 production and selenocysteine-tRNA modification, but this evidence largely comes from cancer and metabolic models rather than BBB-specific systems (134). Fluvoxamine was recently reported to attenuate BBB disruption in drug-resistant epilepsy and inhibit ferroptosis via the sigma-1 receptor–TAMM41 pathway in bEnd.3 cells, making it more directly relevant to BMEC biology than many other repurposed agents (126). Dipyridamole has been identified as a ferroptosis inhibitor in systemic organ injury models, and DPP4-regulated endothelial ferroptosis has been implicated in atherosclerosis progression (135, 136). These findings are valuable as mechanistic leads, but they should be classified as limited or non-CNS endothelial evidence unless BBB-specific and BMEC-specific validation is available.

Accordingly, repurposed drugs should be organized by evidence strength rather than listed as equivalent therapeutic options. Agents with BMEC or BBB readouts can be highlighted as higher-priority candidates, whereas agents supported mainly by non-CNS endothelial or general ferroptosis evidence should be described as hypothesis-generating.

### 6.3 BMEC-specific targeting and endothelial precision strategies

A major translational challenge is that anti-ferroptotic drugs may not reach the injured cerebrovascular endothelium at sufficient concentrations without causing systemic off-target effects. Targeted delivery systems are therefore important for improving the feasibility of endothelial ferroptosis therapy. In stroke models, targeted delivery of ferroptosis inhibitors has been used to reduce recombinant tissue plasminogen activator-associated hemorrhagic transformation (100). BBB-targeted lipid nanoparticles have also improved the neuroprotective effect of ferrostatin-1 against cerebral ischemic damage in experimental stroke (137). These studies support the concept that delivery strategy can determine the therapeutic value of ferroptosis inhibitors.

BMEC-specific targeting can be approached through several complementary strategies. First, receptor-associated delivery may improve endothelial access at the BBB (138). TfR1-related strategies are particularly relevant because BMECs participate in iron exchange between the circulation and the CNS (139, 140). Rosmarinic acid-loaded liposomes provide an example in which drug stabilization, brain delivery, and TfR1-related BMEC targeting were combined to suppress ferroptosis in ischemic brain (12). However, TfR1 is also a physiological iron-transport receptor, so receptor-mediated delivery should be designed to improve endothelial exposure without excessively disrupting CNS iron homeostasis (141, 142).

Second, disease-activated vascular targeting may help concentrate anti-ferroptotic cargo at injured cerebrovascular segments (143). In ischemia/reperfusion injury, thrombolysis-associated BBB damage, traumatic brain injury, or inflammatory vascular injury, activated cerebrovascular endothelium may display altered adhesion, oxidative stress, permeability, and surface-recognition features (144, 145). Ligand-, peptide-, antibody-, or nanoparticle-based platforms could therefore be designed to preferentially accumulate at activated or injured endothelium (146). However, increased brain accumulation alone is insufficient to prove BMEC-specific targeting; endothelial colocalization and BMEC ferroptosis suppression should be demonstrated directly.

Third, pathology-responsive delivery systems may improve spatial and temporal specificity. Carriers that respond to oxidative stress, hypoxia, inflammatory proteases, or acidic microenvironments may release anti-ferroptotic cargo preferentially in injured neurovascular regions (147, 148). Such approaches may be especially relevant when ferroptotic endothelial stress is restricted to ischemic, inflamed, or mechanically injured vascular segments (9, 10). Nevertheless, microenvironment-responsive release should be paired with BMEC-specific validation because activation within diseased brain tissue does not necessarily mean direct endothelial ferroptosis targeting.

Nanocarriers may also improve the utility of agents with poor pharmacokinetic or BBB penetration profiles. DFO-based nanoparticles and DFO-derived mesoporous silica nanoplatforms illustrate how formulation can enhance chelation-based approaches (121). Rosmarinic acid-loaded liposomes provide another example, because this approach combines drug stabilization, brain delivery, and TfR1-related BMEC targeting (12). Similarly, brain-targeted ursolic acid nanoparticles have been developed for anti-ferroptosis therapy in subarachnoid hemorrhage, although this evidence is more focused on neuronal ferroptosis than BMEC-specific ferroptosis (149).

Cell-derived extracellular vesicles represent a second delivery-related strategy. Pericyte-derived exosomal miR-210 improved mitochondrial function and inhibited lipid peroxidation in vascular endothelial cells after traumatic spinal cord injury, suggesting that neurovascular-unit cell communication may be harnessed therapeutically (150). Engineered anti-ferroptosis exosomes targeting M2 microglia have also improved neurological function in ischemic stroke models (151). These extracellular-vesicle approaches are promising because they intersect with the immune–vascular framework discussed above, but their endothelial specificity, cargo stability, biodistribution, immunogenicity, and manufacturing reproducibility remain major barriers.

Evidence of BMEC-specific targeting should include endothelial colocalization, isolated microvessel or single-cell validation, BMEC ferroptosis readouts, BBB functional assays, inflammatory or immune-cell readouts, and rescue experiments. Without this combination, it remains difficult to determine whether a treatment protects the BBB by suppressing endothelial ferroptosis specifically or by acting on neurons, glia, immune cells, or systemic inflammation.

### 6.4 Clinical biomarkers and monitoring strategies

Clinical translation will require biomarkers that can support patient stratification, treatment monitoring, and mechanism-oriented validation. At present, no single clinical biomarker can establish BMEC ferroptosis. General ferroptosis markers, including lipid ROS, malondialdehyde, 4-hydroxy-2-nonenal, GPX4, SLC7A11, ACSL4, and iron-related readouts, are useful but do not identify the cellular source of ferroptosis unless combined with endothelial localization or BBB functional evidence (10, 11, 23, 57). Clinical biomarker studies in acute ischemic stroke suggest that ferroptosis-related signatures may be associated with disease severity or outcome, but such measurements cannot determine whether ferroptosis occurs primarily in BMECs, neurons, glia, or infiltrating immune cells (152).

A more practical approach is to use multimodal biomarker panels rather than single markers. Circulating brain microvascular endothelial cells and endothelial microparticles may reflect BBB-associated endothelial injury or vascular activation, whereas lipid peroxidation signatures such as malondialdehyde, 4-hydroxy-2-nonenal, oxidized lipid species, and lipid ROS-related readouts may indicate ferroptosis-compatible oxidative lipid damage (153–155). Imaging-based readouts, including quantitative PET permeability imaging and dynamic contrast-enhanced MRI, can provide spatial and temporal information about BBB permeability but do not identify ferroptosis as the causal mechanism by themselves (63, 156). Therefore, the most informative monitoring strategy would combine endothelial-source markers, ferroptosis-compatible molecular readouts, BBB permeability imaging, and disease-stage information.

These biomarkers should be interpreted as tools for stratification and treatment-response monitoring rather than as stand-alone proof of BMEC ferroptosis. For example, circulating endothelial markers may indicate vascular injury (154), lipid peroxidation signatures may indicate ferroptosis-compatible oxidative stress (6, 157), and imaging-based BBB permeability correlates may indicate barrier dysfunction (156, 158). Only when these readouts are combined with cell-source information, disease timing, and therapeutic rescue evidence can they help support a mechanistic interpretation of endothelial ferroptosis at the BBB.

### 6.5 Physiological risks of systemic or prolonged ferroptosis blockade

Although ferroptosis inhibition may protect injured BMECs in acute BBB injury, systemic or prolonged suppression of ferroptosis-related pathways may carry physiological risks. Ferroptosis is not only a pathological cell-death mechanism; it is also linked to immune-cell function, inflammatory regulation, host defense, antitumor immunity, redox signaling, and tissue stress responses (159–161). In addition, iron handling, lipid-peroxide detoxification, GPX4 activity, Nrf2 signaling, and TfR1-mediated transport are not disease-specific processes but core components of normal endothelial, immune, and metabolic homeostasis (162, 163). Broad ferroptosis blockade could therefore obscure normal immune responses, interfere with tissue remodeling, or disturb CNS iron and redox balance, especially if treatment is prolonged or systemic.

These considerations do not argue against ferroptosis-targeted therapy. Rather, they indicate that endothelial ferroptosis therapy should aim for transient, disease-stage-specific, and vascular-targeted modulation instead of chronic, nonselective ferroptosis suppression. Acute ischemia/reperfusion injury may require short-window suppression of lipid peroxidation and BBB leakage, whereas chronic neurodegenerative or inflammatory diseases may require more selective modulation of iron handling, immune signaling, and neurovascular repair.

### 6.6 Translational barriers and future priorities

Several barriers currently limit the translation of endothelial ferroptosis-targeted therapies. First, BMEC-specific biomarkers of ferroptosis are not yet established. Most studies rely on general ferroptosis markers such as lipid ROS, malondialdehyde, 4-hydroxy-2-nonenal, GPX4, SLC7A11, ACSL4, or iron accumulation (152, 164, 165). These markers are useful but do not by themselves identify BMEC ferroptosis unless combined with endothelial localization and BBB functional assays (23).

Second, many interventions affect multiple cell types. Iron chelators, Nrf2 activators, selenium compounds, ferroptosis inhibitors, and metabolic drugs can influence neurons, astrocytes, microglia, peripheral immune cells, and endothelial cells simultaneously (166–170). This pleiotropy may be therapeutically useful, but it complicates causal interpretation. Future studies should distinguish endothelial-autonomous effects from broader neuroprotective or anti-inflammatory actions using BMEC-specific genetic models, endothelial-targeted delivery systems, human iPSC-derived BBB models, and spatially resolved molecular profiling (143, 171).

Third, disease timing and context are critical. Acute ischemia/reperfusion injury may require rapid suppression of lipid peroxidation and BBB leakage, whereas AD, PD, chronic hypoperfusion, or sepsis-associated encephalopathy may require long-term modulation of iron handling, immune signaling, and neurovascular repair (156, 172–174). A single anti-ferroptotic strategy is unlikely to fit all disease contexts.

Fourth, delivery remains a central obstacle. BBB disruption can increase drug entry, but relying on passive leakage is unpredictable and may occur too late in the injury process (156, 175). Active targeting to injured endothelium, controlled release, and careful dosing windows will be necessary to reduce off-target effects. At the same time, safety evaluation should consider whether excessive or prolonged suppression of ferroptosis-related pathways may interfere with normal immune defense, antitumor immunity, redox signaling, or tissue remodeling.

**Figure 4:**
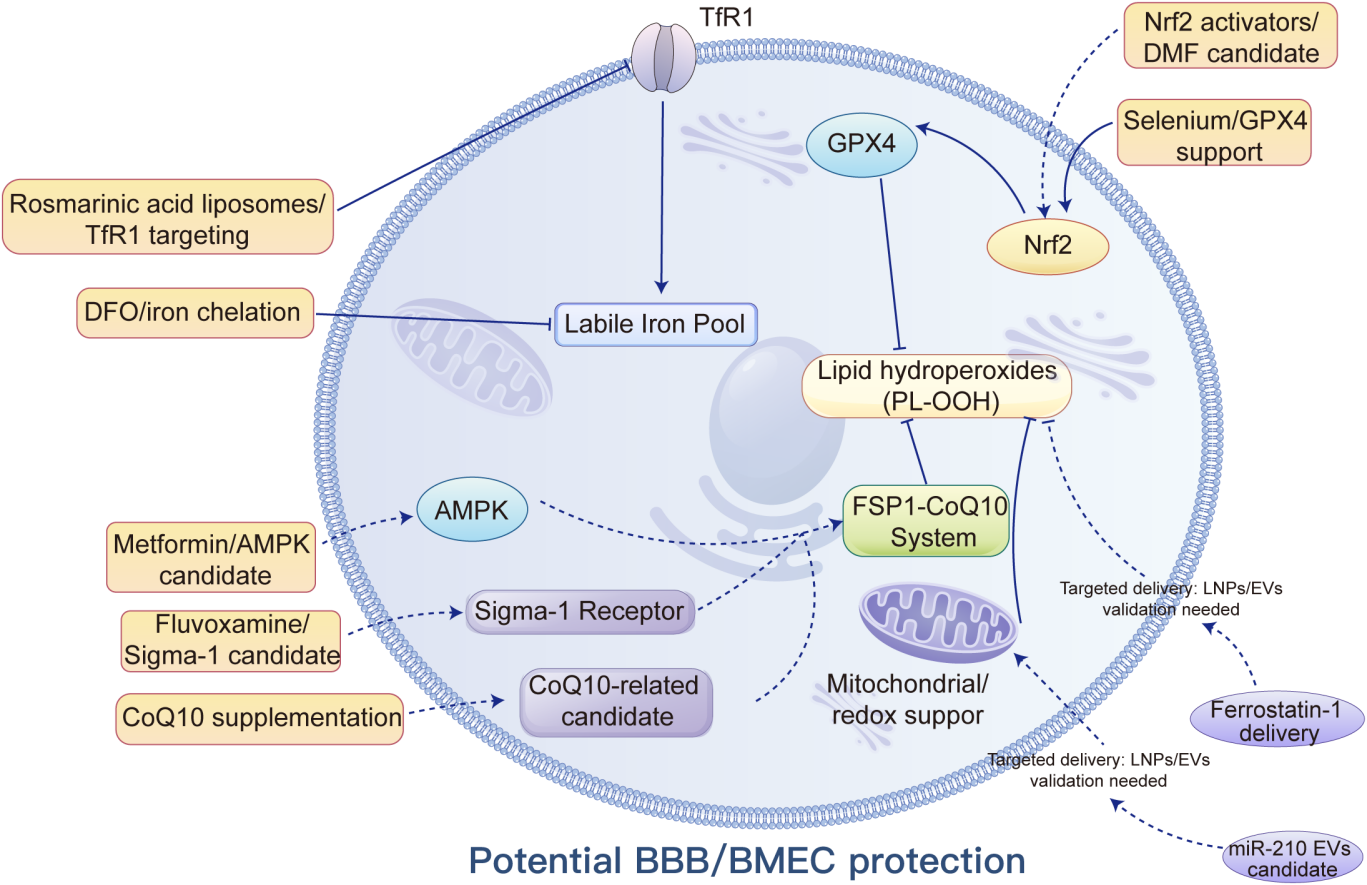
Therapeutic strategies and translational barriers for targeting endothelial ferroptosis at the BBB. Therapeutic strategies aimed at endothelial ferroptosis can be grouped into iron-handling modulation, antioxidant defense enhancement, pathway-based drug repurposing, and targeted delivery approaches. Iron chelation or inhibition of pathological iron uptake may reduce the labile iron pool and limit iron-dependent lipid peroxidation, as illustrated by deferoxamine-based strategies and TfR1-related approaches. Enhancement of endogenous antioxidant defenses, including GPX4- and Nrf2-linked pathways, may improve endothelial resistance to ferroptotic stress in selected BBB injury models. Repurposed agents such as metformin, dimethyl fumarate, and fluvoxamine are shown as pathway-based candidates with different mechanisms and levels of evidence; they should not be interpreted as uniformly BMEC-specific ferroptosis therapies. Targeted lipid nanoparticles, extracellular vesicles, and other delivery platforms may improve drug accumulation at injured neurovascular sites, but their endothelial specificity, biodistribution, timing, safety, and clinical translatability remain unresolved. The image summarizes therapeutic concepts and barriers rather than established clinical interventions. AMPK, AMP-activated protein kinase; BBB, blood–brain barrier; BMEC, brain microvascular endothelial cell; CoQ10, coenzyme Q10; DFO, deferoxamine; DMF, dimethyl fumarate; EVs, extracellular vesicles; FSP1, ferroptosis suppressor protein 1; GPX4, glutathione peroxidase 4; LNPs, lipid nanoparticles; miR-210, microRNA-210; Nrf2, nuclear factor erythroid 2-related factor 2; PL-OOH, phospholipid hydroperoxide; TfR1, transferrin receptor 1.

Taken together, current evidence supports endothelial ferroptosis as a promising therapeutic target for BBB protection, particularly in acute vascular injury models. However, clinical translation will require more than general anti-ferroptotic activity. Future therapeutic development should prioritize BMEC-targeted, time-limited, and biomarker-guided strategies that combine endothelial delivery, BBB functional monitoring, ferroptosis-compatible molecular readouts, immune or inflammatory endpoints, and functional rescue experiments. The major therapeutic strategies and translational barriers for targeting endothelial ferroptosis at the BBB are summarized in **Figure 4**.

## 7 Conclusions and future directions

Endothelial ferroptosis at the BBB is best viewed as an emerging immunovascular mechanism rather than as a fully established linear disease pathway. The strongest current evidence indicates that ferroptosis-related changes in BMECs can accompany hypoxia-induced barrier injury, tight junction protein loss, and BBB dysfunction in selected experimental models (9). Broader BBB and innate-immunity studies further support the concept that endothelial injury, barrier leakage, pattern-recognition receptor signaling, and glial immune activation can interact at the neurovascular interface (13). On this basis, BMEC ferroptosis may help connect vascular redox injury with BBB structural impairment and neuroinflammatory amplification. However, the strength of evidence remains uneven across mechanisms and diseases. BMEC-specific findings should therefore be distinguished from BBB-focused *in vivo* observations, indirect CNS disease evidence, and mechanistic analogies from non-CNS endothelial systems.

Several conclusions emerge from this review. First, BMEC ferroptosis has the most coherent evidence in acute vascular injury settings, particularly hypoxia, ischemia/reperfusion injury, thrombolysis-associated BBB damage, and hemorrhagic transformation (23, 28, 54). In these contexts, iron dyshomeostasis, lipid peroxidation, GPX4/Nrf2-related antioxidant failure, and junctional remodeling can be temporally linked to BBB dysfunction. Second, in chronic neurodegenerative diseases, including Alzheimer’s disease, cerebral amyloid angiopathy, and Parkinson’s disease, the evidence is more indirect and should be interpreted as disease-context or vascular-amplifier evidence rather than definitive BMEC-specific causality. Third, the immunological significance of endothelial ferroptosis lies not only in physical barrier disruption, but also in its potential to promote damage-associated molecular pattern exposure, innate immune sensing, leukocyte trafficking, microglial and astrocytic responses, and inflammatory feedback at the neurovascular interface.

Important limitations remain. A major challenge is the lack of validated BMEC-specific ferroptosis biomarkers. Most studies rely on general ferroptosis markers, including iron accumulation, lipid reactive oxygen species, malondialdehyde, 4-hydroxy-2-nonenal, GPX4, SLC7A11, ACSL4, or mitochondrial morphological changes. These markers are useful, but they do not by themselves establish endothelial cell specificity unless combined with BMEC localization, BBB functional readouts, and rescue experiments. Clinical biomarker studies in acute ischemic stroke suggest that ferroptosis-related signatures may be associated with disease severity or outcome, but such measurements still cannot determine whether ferroptosis occurs primarily in BMECs, neurons, glia, or infiltrating immune cells (152). Candidate clinical readouts, including circulating brain microvascular endothelial cells, endothelial microparticles, lipid peroxidation signatures, and PET- or DCE-MRI-based BBB permeability correlates, may support patient stratification and treatment-response monitoring, but they should not be treated as stand-alone evidence of BMEC ferroptosis. Another challenge is translational relevance. Comparative proteomic analyses show substantial differences among human, non-human primate, mouse brain microvessels, and cultured BMECs, indicating that rodent and *in vitro* models may not fully reproduce human BBB biology (176).

Future work should prioritize cell specificity, temporal resolution, and human relevance. BMEC-selective genetic models, endothelial-targeted delivery systems, spatially resolved lipidomics, redox proteomics, and junctional protein mapping will be needed to determine whether endothelial ferroptosis acts as an initiating trigger, an amplifier, or a downstream consequence of neuroinflammatory injury. Single-cell and spatial transcriptomic approaches can help define how vascular cells, glia, and immune cells interact during ischemia, sepsis-associated encephalopathy, and other neuroinflammatory states (177, 178). Human iPSC-derived systems, brain organoids, and BBB-on-chip platforms may further improve mechanistic validation and therapeutic screening under more human-relevant conditions (179, 180). Therapeutic studies should also move beyond general antioxidant or anti-ferroptotic treatment and instead prioritize BMEC-targeted, time-limited, and biomarker-guided strategies that combine BMEC-specific ferroptosis markers, BBB permeability assays, immune readouts, and functional rescue experiments. Targeted delivery platforms, including TfR1-related BMEC delivery and BBB-directed ferroptosis inhibitor delivery, provide a promising proof of concept strategies, but their safety, timing, biodistribution, and endothelial specificity require further validation before clinical translation (137). Because systemic or prolonged ferroptosis blockade may influence immune defense, antitumor immunity, redox signaling, tissue remodeling, and iron homeostasis, future therapeutic studies should also evaluate treatment duration, disease stage, and physiological safety in addition to BBB protection.

Overall, endothelial ferroptosis provides a useful framework for understanding how vascular redox injury may intersect with BBB dysfunction and neuroinflammatory amplification. The major conceptual advance is not that BMEC ferroptosis explains all BBB pathology, but that it may represent a disease-relevant immune–vascular interface in selected contexts. Establishing this mechanism more firmly will require evidence-stratified studies that integrate BMEC-specific ferroptosis biology, BBB structural and functional assessment, neuroimmune profiling, clinically relevant biomarkers, targeted delivery validation, and translationally relevant human models.
